# Congenital myasthenic syndromes

**DOI:** 10.1186/s13023-019-1025-5

**Published:** 2019-02-26

**Authors:** Josef Finsterer

**Affiliations:** Krankenanstalt Rudolfstiftung, Messerli Institute, Veterinary University of Vienna, Postfach 20, 1180 Vienna, Austria

**Keywords:** Myasthenic syndrome, Myasthenia, Repetitive nerve stimulation, Fatigue, Weakness, Hereditary, Genes, Mutation

## Abstract

**Objectives:**

Congenital myasthenic syndromes (CMSs) are a genotypically and phenotypically heterogeneous group of neuromuscular disorders, which have in common an impaired neuromuscular transmission. Since the field of CMSs is steadily expanding, the present review aimed at summarizing and discussing current knowledge and recent advances concerning the etiology, clinical presentation, diagnosis, and treatment of CMSs.

**Methods:**

Systematic literature review.

**Results:**

Currently, mutations in 32 genes are made responsible for autosomal dominant or autosomal recessive CMSs. These mutations concern 8 presynaptic, 4 synaptic, 15 post-synaptic, and 5 glycosilation proteins. These proteins function as ion-channels, enzymes, or structural, signalling, sensor, or transporter proteins. The most common causative genes are CHAT, COLQ, RAPSN, CHRNE, DOK7, and GFPT1. Phenotypically, these mutations manifest as abnormal fatigability or permanent or fluctuating weakness of extra-ocular, facial, bulbar, axial, respiratory, or limb muscles, hypotonia, or developmental delay. Cognitive disability, dysmorphism, neuropathy, or epilepsy are rare. Low- or high-frequency repetitive nerve stimulation may show an abnormal increment or decrement, and SF-EMG an increased jitter or blockings. Most CMSs respond favourably to acetylcholine-esterase inhibitors, 3,4-diamino-pyridine, salbutamol, albuterol, ephedrine, fluoxetine, or atracurium.

**Conclusions:**

CMSs are an increasingly recognised group of genetically transmitted defects, which usually respond favorably to drugs enhancing the neuromuscular transmission. CMSs need to be differentiated from neuromuscular disorders due to muscle or nerve dysfunction.

## Introduction

**Fig. 1:**
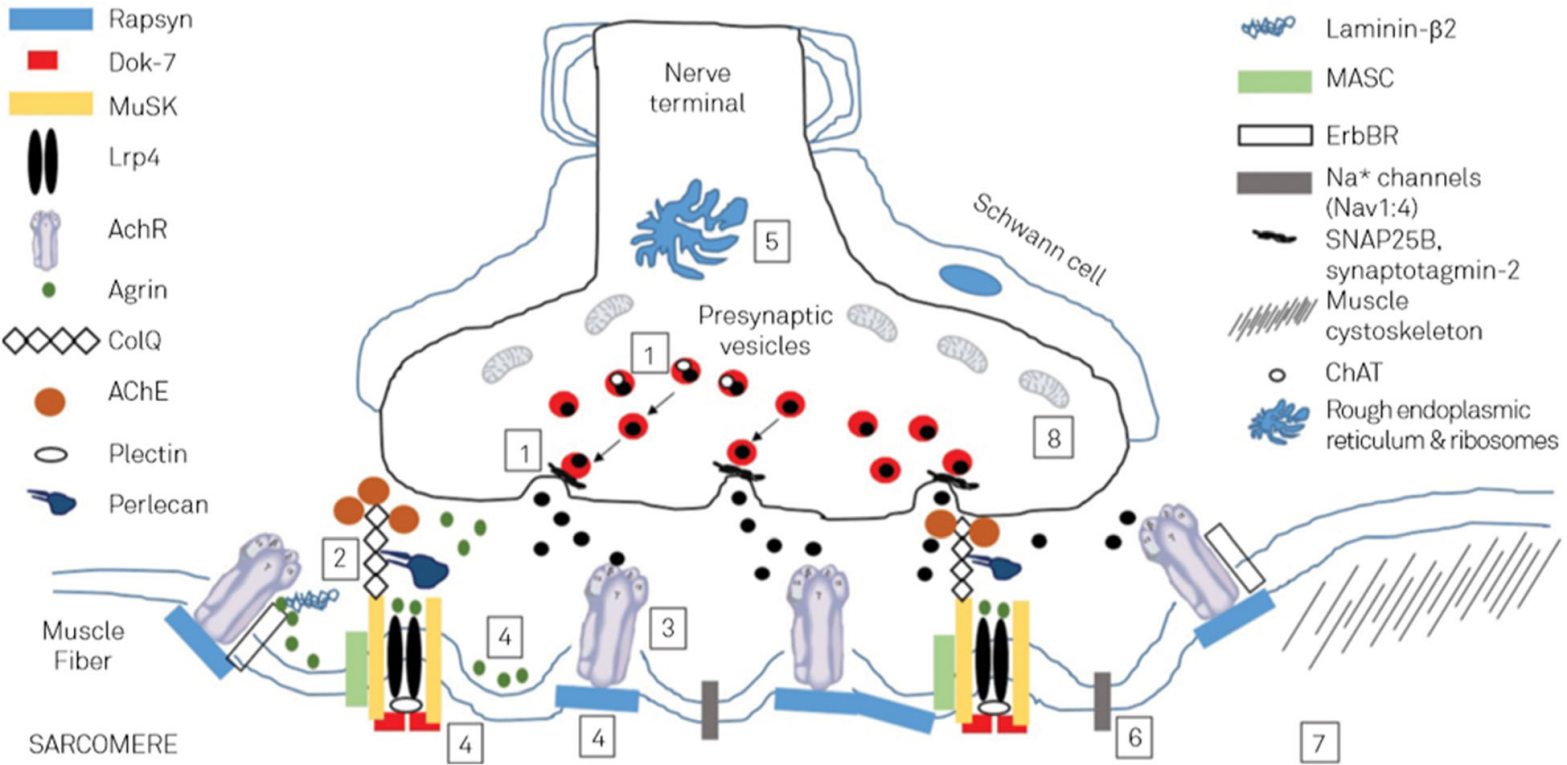
Scheme of the main pathophysiological mechanisms involved in CMS: (1) acetylcholine biosynthesis defects and vesicular transport and fusion defects; (2) AchE deficiency; (3) AchR defects; (4) agrin deficiency; (5) disorders of glycosylation; (6) channelopathies; (7) myopathies with secondary neuromuscular transmission defects; and (8) mitochondrial dysfunction; ChAT: choline acetyltransferase; ErbBR: epidermal growth factor receptor; MASC: muscle-associated specificity component; Lrp4: low-density lipoprotein receptor-related protein 4 [reproduced from Sousa et al. Arq Neuropsquiatr 2016;74:750 [24, 143] [permission applied]

Congenital myasthenic syndromes (CMS) are a heterogeneous group of early-onset genetic neuromuscular transmission disorders due to mutations in proteins involved in the organisation, maintenance, function, or modification of the motor endplate (endplate myopathies) [1, 2] (Fig. 1). CMS are clinically characterised by abnormal fatigability, or transient or permanent weakness of extra-ocular, facial, bulbar, truncal, respiratory, or limb muscles. Onset of endplate myopathy is intrauterine, congenital, in infancy, or childhood, and rarely in adolescence. Severity ranges from mild, phasic weakness, to disabling, permanent muscle weakness, respiratory insufficiency, and early death. All subtypes of CMS share the clinical features of fatigability and muscle weakness, but age of onset, presenting symptoms, and response to treatment vary depending on the molecular mechanism that results from the underlying genetic defect. The term CMS is misleading since not all CMS are congenital.

Aims of the present review were to summarise and discuss previous and recent findings concerning the genotype, phenotype, diagnosis, treatment, and outcome of CMS.

## Methods

Data for this review were identified by searches of MEDLINE for references of relevant articles. Search terms used were “congenital myasthenic syndrome”, “endplate”, or “mutation”, combined with all gene names so far associated with CMS. Results of the search were screened for potentially relevant studies by application of inclusion and exclusion criteria for the full texts of the relevant studies. Randomized controlled trials (RCTs), observational studies with controls, case series, and case reports were included. Only original articles about humans, and published between 1966 and 2017 were included. Reviews, editorials, and letters were not considered. Reference lists of retrieved studies were checked for reports of additional studies. Websites checked for additional, particularly genetic information and for assessing the pathogenicity of CMS mutations were the following:

Neuromuscular homepage: https://neuromuscularwu.stl.edu/

Genetics home reference: https://ghr.nlm.nih.gov/condition/congenital-myasthenic-syndrome

National Organisation for Rare Disorders: https://rarediseases.org/rare-diseases/congenital-myasthenic-syndromes/.

## Results

### History

The first case of a patient with CMS was reported in 1977 by Engel et al. [3]. The first mutation associated with CMS was reported in the *CHRNE* gene by Gomez et al. in 1995 [4]. The first molecular genetic defect resulting in a presynaptic congenital myasthenic syndrome has been reported by Ohno in 2001 [5]. Detection dates of mutations in any of the 32 CMS genes reported in the literature are listed in Table 1.

**Table 1:** First reports of mutations in any of the 32 CMS genes [142]

Gene	Year of first report	NOPR#	Reference for 1st description
COLQ	1998	> 115	[136]
CHRNE	2000	97	[60]
CHAT	2001	46	[5]
SCN4A	2003	3	[96]
RAPSN	2003	84	[137]
MUSK	2004	9	[138]
CHRND	2006	4	[58]
CHRNG	2006	17	[68]
DOK7	2006	> 50	[139]
CHRNA1	2008	6	[54]
LAMB2	2009	1	[49]
AGRN	2009	12	[140]
CHRNB1	2010	3	[10]
PLEC1	2010	3	[104]
GFPT1	2011	29	[141]
DPAGT1	2012	12	[124]
ALG2	2013	5 + 1 family	[108, 122]
ALG14	2013	7	[108]
LRP4	2014	3	[90, 92]
PREPL	2014	1	[93]
SNAP25	2014	1	[30]
SYT2	2014	2 families	[33]
SLC25A1	2014	3	[107]
COL13A1	2015	3	[52]
GMPPB	2015	13	[109]
SLC18A3	2016	3 families	[29]
SLC5A7	2016	7 families	[21]
MYO9A	2016	3	[86]
MUNC13–1	2016	1	[36]
VAMP1	2017	4	[31]
LAMA5	2017	1	[51]
SYB1	2017	1	[7]

NOPR: number of patients/families reported so far, #: since first description (some figures may be imprecise since reporting of patients may overlap between publications)

### Classification

CMS may be classified according to various different criteria. According to the mode of inheritance, CMS may be classified as autosomal dominant (AD), autosomal recessive (AR), as de novo, or as either AD or AR [6]. CMS can be also classified according to the mutated protein (Table 2). Following this classification criterion, 32 different types of CMS can be currently differentiated (Table 2). A third scheme differentiates CMS due to a presynaptic, synaptic or post-synaptic pathology. A fourth category refers to CMS due to glycosylation defects. Furthermore, CMS may be classified according to the function of the mutated protein (e.g. enzyme, structural protein, pore protein). Another possibility to classify CMS is the type of mutation such as point mutations (missense or truncating (frameshift, splice site, nonsense)), deletions, duplications, indels, or insertions. According to the long-term course, CMS may be classified as progressive, fluctuating, or regressive [7].

**Table 2:** Genes mutated in CMS

Gene	Chromosome	MOI	LD	OOCM	PCMS (%)^a^
AGRN	1p36.33	AR	post	con, inf	< 1
ALG2	9q22.33	AR	glyc	con, inf	< 1
ALG14	1p21.3	AR	glyc	con, child	< 1
CHAT	10q11.23	AR	pre	con, inf	4–5
CHRNA1	2q31.1	AD/AR	post	con, adult	< 1
CHRNB1	17p13.1	AF/AR	post	inf	< 1
CHRND	2q37.1	AD/AR	post	inf	< 1
CHRNE	17p13.2	AD/AR	post	con, inf, child, adult	50
CHRNG	2q37.1	AR	post	con	uk
COL13A1	10q22.1	AR	post	con	< 1
COLQ	3p24.2	AR	syn	con, inf, child	10–15
DOK7	4p16.3	AR	post	con, inf, child, adol, adult	10–15
DPAGT1	11q23.3	AR	glyc	inf, adol	< 1
GFPT1	2p13.3	AR	glyc	con, inf, child, adult	2
GMPPB	3p21.31	AR	glyc	con	< 1
LAMA5	20q13.33	uk	pre	con	< 1
LAMB2	3p21.31	AR	syn	con	< 1
LRP4	11p11.2	AR	post	con	< 1
MUNC13–1	19	AR	pre	inf	< 1
MUSK	9q31.3	AR	post	con	< 1
MYO9A	15q23	AR	post	con	< 1
PLEC	8q24.3	AR	post	con	< 1
PREP1	2p21	AR	post	con	< 1
RAPSN	11p13-q1	AR	post	con, inf, child, adol, adult	10–20
SCN4A	17q23.3	AR	post	inf	< 1
SLC18A3	10q11.23	AR	pre	con, inf	< 1
SLC25A1	22q11.21	AR	post	inf	< 1
SLC5A7	2q12.3	AD	pre	con, inf	< 1
SNAP25	20p12.2	AD	pre	con	< 1
SYB1	12p	uk	pre	inf	< 1
SYT2	1q32.1	AD	pre	child	< 1
VAMP1	12p13.21	AR	pre	con	< 1

*MOI* mode of inheritance, *LD* localisation of defect, pre: presynaptic, syn: synaptic, post: post-synaptic, glyc: glycosylation defect, *OOCM* onset of clinical manifestations, *con* congenital, *inf* infantile, *child* childhood, *adol* adolescence, adult: adulthood *PCMS* prevalence of various subtypes, ^a^: according to [6], *uk* unknown

### Frequency

Concerning the frequency of CMS only limited data are available since most of the current knowledge has been obtained by reports of isolated cases [8]. According to a recent review, the prevalence of CMS is estimated as 1/10 that of myasthenia gravis, which is 25–125/1000000 [6]. In a recent study on the frequency of autoimmune myasthenia and genetic myasthenia in patients under 18y of age, the prevalence of CMS in Great Britain was calculated as 9.2/1000000 but varies considerably between the regions between 2.8 and 14.8/1000000 [9]. In the Brasilian state of Parana the prevalence of CMS was estimated as 0.18/100000 [10]. Most likely, these prevalence figures are underestimations because CMS may go undetected if mixed up with one of the many differential diagnoses or if manifesting only with mild symptoms. In several regions worldwide local increases of certain mutations have been detected. In the Roma population of South-East Europe an increased frequency of the c.1327delG variant in the *CHRNE* gene has been reported [11]. Similarly, an increased prevalence of the variant c.1353duplG in the *CHRNE* gene has been reported in Algeria and Tunisia [12]. In Spain and Portugal the *CHRNE* variant c.130dupC is highly prevalent. *CHRNE*-related CMS is generally regarded as the most common of the CMS. In Western or central Europe the *RAPSN* variant c.264C > A and the *DOK7* variant c.1124_1172dupTGCC are highly prevalent. Concerning the frequency of the 32 CMS subtypes, mutations in the *CHRNE* gene are the most frequent, accounting for 30–50% of the CMS cases, a figure which varies significantly between different ethnia [13]. Mutations in the *CHRNE* gene result in acetylcholine-receptor deficiency or abnormal channel kinetics [14]. The second most frequent defect is that in the *RAPSN* gene accounting for 15–20% of the CMS cases. The third and fourth most frequent CMS subtypes are *COLQ* and *DOK7* variants accounting for 10–15% of the CMS cases. Mutations in the *CHAT* gene account for 4–5% of the CMS cases [6]. Mutations in *GFPT1* can be found in 2% of the CMS cases. However, these figures may vary between countries and regions under investigation. In a study of 34 CMS families from Israel the genes most frequently mutated were *RAPSN* (*n* = 13), *COLQ* (*n* = 11), and *CHRNE* (*n* = 7) [15]. All other mutated proteins may contribute with less than 1% of the CMS cases to the general group of CMS. About 75% of the CMS cases are due to mutations in genes that encode different subunits of the acetylcholine receptor (*CHRNA1, CHRNB1, CHRND, CHRNE*) or proteins important to maintain the structure or function of the NMJ, such as *MUSK*, *RAPSN* or *DOK7* [16, 17]. The most common causative genes are *CHAT, COLQ, RAPSN, CHRNE, DOK7*, and *GFPT1*.

### Mutated proteins

Currently, 32 proteins located on the presynaptic, synaptic or post-synaptic part of the motor endplate/neuromuscular junction (NMJ) or proteins undergoing abnormal glycosylation have been reported to be involved in the various types of CMS. Eight proteins are associated with presynaptic CMS, four with synaptic CMS, fifteen with post-synaptic CMS, and five with glycosylation defects. Proteins affected in CMS have different functions, such as ion channels (AchR, SNC4A), structural proteins (LAMA5, COL13A1, RAPSN, PLEC, COLQ), signalling molecules (AGRN, LRP4, MUSK, DOK7), catalytic enzymes (CHAT, GFPT1, DPAGT1, ALG14, ALG2, GMBBP, PREPL, SLC25A1), sensor proteins (SYT2), or transport proteins (SLC18A3) [18].

#### Pre-synaptic CMS

The majority of CMS is caused by defects in post-synaptic proteins but some of the CMSs are also caused by defects of the presynaptic proteins [19]. These include the proteins SLC5A7, CHAT, SLC18A3, SNAP25, VAMP1, SYB1, SYT2, and MUNC13–1 [1, 6]. Presynaptic defects may be further categorised as disorders affecting the axonal transport, disorders affecting the synthesis and recycling of acetylcholine, and disorders affecting the exocytosis of synaptic vesicles.

#### Disorders affecting the axonal transport

##### SLC5A7

Recently, mutations in the presynaptic, Na-dependent, high-affinity choline transporter-1 (CHT) encoded by the *SLC5A7* gene have been identified as a rare cause of CMS [20]. Mutations in this gene also cause allelic AD forms of distal motor neuropathy [20]. Patients with *SLC5A7*-related CMS present with severe muscle weakness, ranging from lethal antenatal arthrogryposis and severe hypotonia to a neonatal form of CMS with episodic apneas. The prognosis of apneas is more favourable if patients respond to AchEI [20]. In another family, patients presented with severe neuro-developmental delay with cerebral atrophy [21]. Low-frequency repetitive nerve stimulation (LF-RNS) usually shows a decrement but sometimes only after previous high-frequency RNS (HF-RNS) during 10s with 20 Hz [20]. All patients reported responded favourably to AchEI and one patient also to salbutamol [20].

#### Disorders affecting the synthesis and recycling of acetylcholine

##### Chat

The *CHAT* gene encodes for the cholin acetyltransferase, which promotes the resynthesis of acetylcholine [22]. Clinically, patients present with ptosis, limb muscle weakness, easy fatigability, and recurrent episodes of potentially fatal apnea [22]. Episodes of apnea have an abrupt onset but may be triggered by physical or emotional stress or acute illness. Cerebral hypoxia/ischemia during apneic episodes may secondarily result in global developmental delay with delayed myelination and signs of hypoxic-ischemic injury on cerebral imaging [23]. Apnea may be present already at birth or may rarely begin during childhood or early adulthood [24]. Infections or stress may lead to life-threatening failure of neuromuscular transmission [25]. Muscle MRI is usually normal [26]. Ultrastructural investigations of the NMJ may be non-informative [22]. In-vitro microelectrode studies performed in biopsied muscle may show moderate reduction of quantal release [22]. AchEI may be beneficial for mild symptoms [27] but may not prevent the occurrence of apneic episodes [23]. Some patients may require permanent ventilation [25]. Despite the application of AchEI, permanent proximal muscle weakness may develop and may lead to wheelchair-dependency [28].

##### SLC18A3

The *SLC18A3* gene encodes the vesicular acetylcholine transporter VAchT [19]. VAchT loads newly synthesised acetylcholine from the neuronal cytoplasm into synaptic vesicles [19, 29]. *SLC18A3*-related CMS have been reported in only three families [19, 29]. Index cases from the first two families presented with ptosis, ophthalmoparesis, fatigue, weakness, and apneic crises [29]. Interestingly, muscle manifestations in these patients deteriorated in cold water (paramyotonia) [29]. One of the patients also had learning difficulties and left ventricular systolic dysfunction [29]. The two patients from family 3 presented with respiratory failure since birth requiring mechanical ventilation [19]. Index patients of family 1 and 3 showed prominent decrement on LF-RNS followed by a prolonged period of postactivation exhaustion [29]. In one patient the decremental response could be unmasked only after isometric contraction, a well-recognised feature of presynaptic disease [29]. AchEI were only moderately effective.

#### Disorders affecting the exocytosis of synaptic vesicles

##### SNAP25

*SNAP25* encodes a “soluble *N*-ethyl-maleimide sensitive fusion (NSF) attachment” (SNARE) protein essential for exocytosis of synaptic vesicles from nerve terminals and of dense-core vesicles from endocrine cells [30]. Ca++ − triggered exocytosis is initiated when synaptobrevin, attached to synaptic vesicles (v-SNARE), assembles with SNAP25B and syntaxin, anchored in the presynaptic membrane (t-SNAREs) into an α-helical coiled-coil, held together by hydrophobic interactions [30]. Mutations in the *SNAP25* gene result in inhibition of synaptic vesicle exocytosis [30]. *SNAP25*-related CMS has been reported only in a single female who presented with myasthenia, congenital contractures, cortical hyperexcitability, cerebellar ataxia, and severe intellectual disability [30]. In this patient neuromuscular transmission was compromised due to reduced quantal release.

##### VAMP1

The *VAMP1* gene encodes for a presynaptic protein, which is crucial for vesicle fusion at the presynaptic membrane [31]. So far, *VAMP1*-related CMS has been reported in a Kuwaiti and an Israeli family [31]. The two patients from the Kuwaiti family presented shortly after birth with hypotonia, muscle weakness, feeding difficulties requiring gavage feeding, delayed motor development, and ophthalmoparesis [31]. One patient had joint contractures [31]. The two patients from the Israeli family presented with severe congenital hypotonia and muscle weakness, feeding difficulties requiring percutaneous entero-gastrostomy (PEG) implantation, and severely delayed developmental milestones [31]. One of them additionally had joint laxity and kyphoscoliosis, the other one had knee contractures and respiratory insufficiency [31]. Both patients were unable to generate antigravity postures or movements [31]. Electrophysiologic examination revealed severely low compound muscle action potentials (CMAPs) and presynaptic impairment [31]. Both Israeli patients profited from pyridostigmine [31].

##### SYB1

*SYB1* encodes for the SNARE protein synaptobrevin, which is essential for synaptic vesicle exocytosis [7]. Mutations in *SYB1* have been reported in a single patient with CMS [7]. The female presented at birth with marked hypotonia and feeding difficulties [7]. At age 2y, severe muscle weakness, wasting, and mild ophthalmoparesis developed [7]. LF-RNS was followed by a decremental response, and 20 Hz stimulation during 5 s increased the CMAP-amplitude up to 9-fold [7]. Pyridostigmine had a moderate beneficial effect [7]. Over the years, muscle weakness slightly improved such that she could sit unaided but slurred speech and inability to swallow persisted [7]. She died from respiratory failure precipitated by infection at age 14y [7].

##### SYT2

*SYT2* encodes the presynaptic protein synaptotagmin that interacts with SNAP25 and is involved in the calcium-evoked acetylcholine release. *SYT2*-related CMS has been reported in two families [32, 33]. Clinically, patients presented with prominent muscle weakness of the lower limbs and areflexia. Motor neuropathy has been another phenotypic feature. Various family members presented with foot deformities (pes cavus (hollow feet), hammertoes, pes planus, clawing), hyperlaxity, hip dysplasia, hypotonia, diffuse limb weakness and wasting, and mild ptosis [32]. LF-RNS evoked a decremental response in several family members. Maximal voluntary contraction for 10s (facilitation) resulted in a marked increase of the CMAP [32]. 3,4-DAP was more effective than pyridostigmine [32].

##### MUNC13–1

MUNC13–1 acts as a master regulator of neurotransmitter release, mediating docking-priming of synaptic vesicles and various presynaptic plasticity processes [34]. MUNC13–1 bridges the vesicle and plasma membranes from the periphery of the membrane-membrane interface [34]. In the inactive state, MUNC13–1 locks syntaxin, another SNARE protein, in a folded state [35]. Upon Ca2+ entry into the nerve terminal, MUNC13–1 unlocks syntaxin by displacing MUNC18 allowing syntaxin to interact with synaptobrevin and SNAP25B to effect vesicle exocytosis [35]. Mutations in *MUNC13–1* have been reported only in a single patient. In a 2yo girl with generalised hypotonia, feeding difficulties, respiratory insufficiency, microcephaly, callosal atrophy, facial dysmorphism, variable ptosis, quadruparesis, scoliosis, flexion contractures, and paroxysmal EEG activity, whole exome sequencing (WES) revealed the homozygous mutation c.304C > T in the *MUNC13–1* gene [36]. The CMAP was low at rest, and LF-RNS revealed a decrement of 20–40% and HF-RNS an increment between 0.8 and 4 mV [36]. Pyridostigmine and 3,4-DAP were only partially effective [36].

##### Synaptic CMS

Four of the 32 subtypes of CMS are due mutations in genes encoding synaptic proteins. These include COLQ, LAMB2, LAMA5, and COL13A1 [1].

##### COLQ

*COLQ* encodes a multidomain functional protein of the NMJ, crucial for anchoring AChE to the basal lamina and accumulating AChE at the NMJ [37]. *COLQ*-related CMS may not only be due to point mutations, deletions or duplications, but also due to copy number variant mutations (deletion or duplication of the entire gene) [21]. Mutations in *COLQ* cause AchE deficiency. Clinically, *COLQ*-related CMS presents with a broad range of features and severity from mild muscle manifestations, such as gait disturbance with independent ambulation and mild respiratory insufficiency, to wheel-chair boundness, or early death [38]. Usually, however, the clinical manifestations are severe. Particularly the axial muscles can be severely affected and the ocular muscles are usually spared [1]. Several patients with a limb-girdle muscular dystrophy (LGMD)-like phenotype have been reported [39]. Some patients may experience short- or long-term relapses, being triggered by AchEI, infections, puberty, or pregnancy [40]. Occasionally, the phenotype includes ptosis, ophthalmoparesis, or facial diplegia. The pupillary response may be slowed. Some patients may present with respiratory failure at birth or later in the course [41, 42]. Some patients may present with severe scoliosis [43]. In two patients, isolated vocal cord paralysis has been reported as initial manifestation, which did not respond to pyridostigmine, mildly to 3,4-DAP, but favourably to ephedrine [42]. Rarely, microcephaly has been reported [41]. Interestingly, heterozygote carriers can present with congenital ptosis [44]. Single nerve stimuli may evoke double responses. Muscle MRI may be normal [26]. Muscle biopsy may show mild fibre-size variation and marked type-I-muscle fiber predominance [45]. Some patients may show dystrophic features and dystrophin deficiency [45]. Biochemical investigations may reveal complex-I-deficiency [45]. Pyridostigmine is ineffective or even detrimental [46]. However, several patients have responded favourably to ephedrine [3, 47] and some to salbutamol [48].

##### LAMB2

The *LAMB2* gene encodes for the laminin-beta-2 protein, which plays a major role in the development of the NMJ. The gene is ubiquitously expressed but manifests mainly at the NMJ. *LAMB2*-related CMS has been reported only in a single 22yo female so far [49]. The patient presented clinically with episodes of respiratory distress, delayed motor milestones, and persistently constricted pupils and nephrotic syndrome (Pierson syndrome), requiring kidney transplantation [49]. Later in the course, the patient developed ptosis, ophthalmoparesis, and scoliosis [49]. LF-RNS was decremental, which became more pronounced at 10 Hz stimulation [49]. Microelectrode recordings revealed profound reduction of the quantal content of endplate potentials [49]. AchEI caused deterioration, such that the patient required ventilatory support [49]. On the contrary, the patient responded favourably to ephedrine [49].

##### LAMA5

The *LAMA5* gene encodes for the protein laminin-A5 involved in the maintenance and function of the extracellular matrix [50]. Laminin-A5 is a major component of the basal membrane and cooperates with growth factors and matrix-dependent receptors in cell proliferation and differentiation [50]. *LAMA5*-related CMS has been reported only in a single patient [51]. The female presented at age 24y with muscle weakness, myopia, and facial tics [51]. Cerebral MRI showed mild volume loss and periventricular T2-hyperintensities [51]. LF-RNS evoked a decrement of 55% but a 250% increment after 10s of maximal contraction [51]. Endplate studies identified profound reduction of the endplate potential quantal content and endplates with normal post-synaptic folding that were denervated or innervated by small nerve terminals [51].

##### COL13A1

The *COL13A1* gene encodes the α-chain of an atypical non-fibrillar collagen with a single transmembrane domain [52]. COL13A1 is localised to the NMJ, where it is responsible for clustering of the AchR during myotube differentiation [52]. Mutations in this gene manifest clinically as CMS, which has been reported in three patients (2 females, 1 male) from two families [52]. Two of these patients manifested with congenital respiratory insufficiency, bulbar weakness, or facial weakness. All three patients presented with feeding difficulties, ptosis, limb weakness, and dysmorphism [52]. Two patients each presented with spinal stiffness or distal joint laxity, and one patient with ophthalmoparesis and cognitive impairment. Two showed a decremental response to RNS and two an increased jitter [52]. Two required non-invasive positive pressure ventilation (NIPPV). In two patients pyridostigmine was ineffective. Salbutamol respectively 3,4-DAP was beneficial [52].

#### Post-synaptic disorders

Fifteen of the CMS subtypes are due to mutations in genes encoding post-synaptic proteins. These include CHRNA1, CHRNB1, CHRND, CHRNE, CHRNG, DOK7, MUSK, MYO9A, AGRN, LRP4, PREP1, SCN4A, RAPSN, PLEC, and SLC25A1. Thus, post-.synaptic CMSs represent the vast majority of the CMS subtypes. Post-synaptic CMS are subdivided into primary AchR deficiency, kinetic abnormalities of the AChR, and defects within the AchR-clustering pathway.

#### Primary AchR deficiency

##### CHRNA1

The *CHRNA1* gene encodes the α-subunit of the nicotinergic, post-synaptic AchR. *CHRNA1* mRNA undergoes alternative splicing and two splice variants (P3A- and P3A+) are produced [53]. Mutations in *CHRNA1* result in imbalance between the two splice variants with an increase in P3A+. *CHRNA1* mutations reduce the number of AchR at the post-synaptic membrane [54]. The pattern of inheritance is AD if *CHRNA1* mutations cause a slow channel CMS (SCCMS) or AR in case of primary AchR-deficiency [54]. The first *CHRNA1*-related CMS were reported in 2008 [54] (Table 1). Patients presented already prenatally with growth retardation, reduced movements, edema, contractures, and postnatally with dysmorphism, muscle wasting, scoliosis, contractures, and pterygia [55]. Concerning the frequency of *CHRNA1* mutations, they were found only in a single of 18 Brasilian families with CMS [10]. *CHRNA1*-related CMS seems to respond favourably to AchEI [54]. Antisense oligonucleotides (AONs) have been shown to restore the balance between the two splice variants and are thus expected to be beneficial in patients carrying such mutations [53].

##### CHRNB1

The *CHRNB1* gene encodes for the β-subunit of the nicotinergic, post-synaptic AchR. The first mutations in *CHRNB1* causing CMS were reported in a Brazilian study in 2008 [10] (Table 1). The first patient published was a 28yo male manifesting since birth with ptosis, ophthalmoparesis, dysphagia, proximal limb muscle weakness, scapular winging, weakness of axial muscles, wasting, and scoliosis [10]. He showed a decremental response to RNS, had double discharges, and a myopathic EMG. The course was progressive but he benefitted from fluoxetine [10]. The second patient carrying a *CHRNB1* mutation was a 3wo male manifesting with ptosis, facial weakness, severe hypotonia, and respiratory insufficiency requiring assisted ventilation [56]. The response to LF-RNS was decremental. AchEI were ineffective and he was put on quinidine but was lost to follow-up [56]. In a Spanish study of a CMS cohort, a third patient with a *CHRNB1* mutation was identified but no clinical details were provided [57].

##### CHRND

The *CHRND* gene encodes the δ-subunit of the nicotinergic, post-synaptic AchR. The first mutation in *CHRND* causing CMS was reported in a German patient with early-onset CMS manifesting with feeding difficulties, moderate, generalised weakness, and recurrent episodes of respiratory insufficiency provoked by infections [58]. The second patient was a 20yo female with moderate to severe myasthenic manifestations since birth [59]. She had a marked decremental response to LF-RNS. She responded poorly to AchEI but clearly to 3,4-DAP [59]. One of her siblings with a similar presentation had died at age 11 m [59]. Two further patients were reported in a study of CMS patients from Israel but no clinical details were provided [15].

##### CHRNE

The *CHRNE* gene encodes for the ε-subunit of the AchR. The first mutation in the *CHRNE* gene causing a CMS has been reported already in 2000 (Table 1) [60]. Since then various different types of mutations have been reported and it is estimated that up to half of the patients with a CMS carry a *CHRNE* mutation, thus representing the gene most frequently mutated in CMS [6]. In a study of 64 CMS patients from Spain, *CHRNE* mutations were detected in 27% of the patients [57]. In a study of 45 patients from 35 Israeli CMS families, *CHRNE* mutations were found in 7 kinships [15]. In a study of 23 families with CMS from Maghreb countries, the founder mutation c.1293insG was found in 60% of these patients [61]. Type and severity of clinical manifestations of *CHRNE* mutations may vary considerably between affected families. Some patients may present with only ptosis whereas others may present with severe generalised myasthenia [62]. Most patients present at birth with mildly progressive bulbar, respiratory, or generalized limb weakness with ptosis or ophthalmoplegia [63, 64]. Single patients may die prematurely in infancy from respiratory failure [65]. Some patients may have myasthenic symptoms since birth and achieve ambulation late or not at all [65]. Single patients present with a fluctuating course [57]. Single patients develop severe scoliosis [27]. RNS may be decremental [27] or may be normal [64]. Single-fiber EMG (SF-EMG) may reveal an increased jitter [64]. Some patients may show repetitive CMAPs [27]. Most patients respond favourably to AchEI [61]. However, in some patients pyridostigmine and 3,4-DAP may be ineffective or may worsen the phenotype. Albuterol can be highly effective in single patients [66]. Other patients may profit significantly from salbutamol [14]. Fluoxetine alone may be ineffective but in combination with salbutamol a significant improvement can be achieved [67].

##### CHRNG

The *CHRNG* gene encodes for the fetal γ-subunit of the AchR. Mutations in the *CHRNG* gene cause CMS with multiple ptyerygia (lethal multiple pterygia syndrome (LMPS) or the Escobar variant of multiple pterygia syndrome (EVMPS)) [68]. In a study of seven families with Escobar syndrome (contractions, multiple pterygia, respiratory distress), mutations in the *CHRNG* gene were detected in 12 family members [68]. The female to male ratio was 7:5. Some patients presented with decreased fetal movements, facial weakness, respiratory distress, arthrogryposis, short stature, kyphosis/scoliosis, dysmorphism, high-arched palate, cleft palate, arachnodactyly, or cryptorchism [68]. None presented with myasthenic manifestations postnatally. *CHRNG* mutations may be also responsible for the allelic disease fetal akinesia deformation sequence (FADS) [54]. In a study of 46 CMS patients from Spain, five carried a mutation in the *CHRNG* gene [57]. They all presented with arthrogryposis and delayed motor milestones, and some of them with poor sucking [57]. Interestingly, none of them received drugs usually given for CMS. In a study of three Iranian *CHRNG*-related CMS patients, no drug treatment was applied [69]. One of the patients presented with short neck, mild axillar pterygia, elbows and knees, joint contractures, clenched hands with thumbs held across palm and club feet (varus). The patient had rockerbottom feet, with almost no movement in ankles. Facial dysmorphism included hemangioma over forehead and nose, strabismus, flat nasal bridge, and downturned corners of mouth [69].

#### Kinetic abnormalities of the AChR

According to the kinetics of the AChR, two functionally distinct types of CMS are differentiated, fast channel CMS (FCCMS) and SCCMS.

##### FCCMS

FCCMS is characterized by only a short opening time of the AchR. FCCNS is due to loss of function mutations in subunits of the AchR. These mutations cause abnormally brief AChR channel openings by enhancing the channel closing rate or by curtailing the channel opening rate. [35]. Decrease in AChR affinity for acetylcholine or altered fidelity of channel openings can also cause shortened channel openings [35]. The safety margin of neuromuscular transmission is compromised by the decreased probability of channel openings and by the accelerated decay of the synaptic response [35]. FCCMS usually present in early childhood with an infantile phenotype. FCCMS responds to 3,4-DAP in combination with pyridostigmine [35].

##### SCCMS

SCCMS, on the contrary, is characterized by a prolonged opening time of the AchR. SCCMS are usually due to gain-of-function mutations in AchR subunit genes. In most patients, SCCMS follows an AD trait of inheritance [1]. On the contrary, most primary AchR deficiency syndromes follow an AR trait of inheritance. Mutations in any of the four AChR adult subunits can alter ion channel function of the AchR. Onset of SCCMS subtypes is usually after adolescence with initially mild phenotypes. Only rare cases present in early life and become severely disabled in the first decade [18]. In most patients there is selective, severe involvement of cervical and wrist and finger extensor muscles [35]. Electrophysiological investigations of SCCMS frequently reveal repetitive discharges (a single nerve stimulus evokes repetitive compound muscle action potentials) [1]. Intake of AchEI typically deteriorates the clinical manifestations [1]. SCCMS do not respond to edrophonium.

#### Defects within the AchR-clustering pathway

##### DOK7

The *DOK7* (downstream-of-kinase) gene encodes for the protein DOK7, which is involved in signaling downstream of receptor and non-receptor phosphotyrosine kinases [70]. DOK7 activates MUSK via dimerisation [71]. Various mutations have been reported in the *DOK7* gene. Particularly reported were deletions [72]. They may occur during DNA replication since there is breakpoint microhomology and an inverted repeat [72]. Concerning the frequency of *DOK7*-related CMS, it was the second most frequent subtype in a Brasilian cohort [10]. Clinical onset is characterised by gait disturbance due to muscle weakness after normal motor milestones [73]. Proximal limb muscles are more strongly affected than distal limb muscles (LGMD-like pattern) [73]. Congenital *DOK7*-related CMS may manifest as stridor due to vocal cord paralysis, occasionally requiring intubation and artificial ventilation [74]. Occasionally, patients present with ptosis but only rarely with ophthalmoparesis. Fatigability is often absent but prolonged periods of weakness may occur [75]. Feeding difficulties may require nasogastral tube feeding or even PEG implantation [74]. Muscle biopsy may show lipidosis and defective branching of terminal axons, which results in a unique terminal axon contacting en passant post-synaptic cups [76]. AchEI are usually ineffective and may even worsen clinical manifestations [73]. Ephedrine (initially 25 mg/d and increased to 75-100 mg/d) seems to be an effective alternative [77, 78]. Salbutamol may be effective in *DOK7*-related CMS as well [79]. Single patients profit from albuterol, which can prevent progression of muscle weakness in LGMD-type *DOK7*-related CMS [80].

##### Musk

*MUSK* encodes for a protein that is involved in endplate maturation, maintenance of the endplate functions, proper functioning of rapsyn, and functioning of the AchR [24]. MUSK forms a co-receptor for agrin with LRP4 and induces AchR clustering [18]. CMS due to *MUSK* mutations is rare and manifests as respiratory insufficiency, neonatal ptosis, proximal limb muscle weakness, and weak bulbar, facial, or ocular muscles [18]. A 30yo Chinese male with the LGMD-type of *MUSK*-related CMS developed mild atrophy of the leg muscles [81]. LF-RNS was decremental. Pyridostigmin deteriorated the clinical manifestations [81]. Another male infant manifested with congenital respiratory failure requiring mechanical ventilation, axial weakness with head drop, facial weakness, proximal limb weakness, and ophthalmoparesis [82]. Salbutamol was effective but 3,4-DAP had only a mild effect, and AchEI worsened the phenotype [82]. In a female with congenital hypotonia and respiratory distress requiring mechanical ventilation for 8 m, respiratory distress and nocturnal apnea with vocal cord paralysis recurred at age 8y [42]. 3,4-DAP was effective [42]. In two Turkish brothers *MUSK* mutations manifested as LGMD-type CMS [83]. *MUSK*-related CMS may also manifest as congenital ptosis and later in life with fatigability [84]. In another patient with *MUSK*-related CMS and congenital respiratory insufficiency, albuterol was moderately effective but AchEI, 3,4-DAP, and ephedrine were ineffective [85].

##### MYO9A

The *MYO9A* gene encodes an unconventional myosin [86]. Mutations in the *MYO9A* gene causing CMS have been reported in 3 patients from 2 unrelated families [86]. Patient-1 presented as a neonate with dysphagia requiring PEG-feeding, limb muscle weakness, episodic apnoea, respiratory failure, and ptosis. SF-EMG showed an increased jitter in the orbicularis oculi muscle. The patient responded favourably to a combination of pyridostigmine and 3,4-DAP [86]. Patients-2 and 3 were two Kurdish siblings, both with prenatal onset with reduced fetal movements. At birth patient-2 presented with bilateral ptosis and after 2 months with generalized hypotonia and dysphagia and chewing difficulty. She had delayed motor milestones, symmetric, multivectorial nystagmus, left eye upgaze deviation, and ophthalmoplegia. Respiratory crises could be triggered by 3.4-DAP, fluoxetine, and respiratory infections. Patient-3 presented with bilateral ptosis within the first week after birth, ophthalmoplegia, nystagmus, and oculomotor apraxia, and developed generalized hypotonia, absence of head and truncal control, and difficulty with swallowing and chewing. Sitting was achieved at 12 m, head control at 18 m, and the ability to walk unassisted at 30 m of age. RNS was decremental. Both patients responded favourably to pyridostigmine. The unaffected parents were consanguineous and had previously lost four children during the first year of life, all with respiratory failure, feeding difficulties, and hypotonia [86].

##### AGRN

The *AGRN* gene encodes for a proteoglycan, which is secreted by the terminal nerve into the synaptic cleft. At the post-synaptic membrane agrin binds to the LRP4 receptor to phosphorylate and activate MUSK [24]. Thus, agrin plays a critical role in the development and maintenance of the NMJ [87]. Mutations in the *AGRN* gene manifest phenotypically as either early-onset or late-onset CMS [24]. The infantile-onset type is characterised by weakness and wasting of the lower limbs with fatty replacement of myocytes in the posterior compartment. The late-onset type is characterised by ptosis, ophthalmoparesis, and mild facial and bulbar weakness. Rarely, mutations in the *AGRN* gene may be associated with dropped head syndrome [87]. In a study of 5 patients from 3 families carrying *AGRN* mutations, all presented with permanent distal muscle weakness and wasting in addition to myasthenia [88]. Both types of *AGRN*-related CMS respond favourably to ephedrine. Pyridostigmine and amifampridine were ineffective [24].

##### LRP4

The *LRP4* gene encodes for a protein, which functions as a receptor for agrin [89]. LRP4 forms a complex with MUSK and mediates MUSK activation by agrin [89]. Activated MUSK together with DOK7 stimulates rapsyn to concentrate and anchor AchR at the post-synaptic membrane and interacts with other proteins implicated in the assembly and maintenance of the NMJ [90]. LRP4 is thus essential for pre- and post-synaptic specialisation of the NMJ [91]. The first mutation in the *LRP4* gene causing CMS was reported in 2014 (Table 1) [90]. A newborn female presented with respiratory arrest and feeding difficulties, and required feeding and ventilator support until 6 m of age [90]. Motor milestones were delayed and she developed easy fatigability with temporary wheelchair-dependency [90]. At ages 9 and 14y she presented with ptosis, ophthalmoparesis, and limb weakness [90]. RNS evoked a decremental response, which improved upon application of edrophonium. AchEI worsened the clinical manifestations [90]. A second kinship harbouring *LRP4* mutations was reported in 2015 [92]. The two sisters, aged 34 and 20y, presented with delayed motor milestones, slight chewing and swallowing difficulties, and later developed limb weakness [92]. Albuterol was highly effective [92].

##### PREPL

*PREPL* encodes for an ubiquitously occurring propyl-endopeptidase, with highest levels in the brain, kidneys, and muscle [93]. PREPL acts as an effector of the clathrin-associated adaptor protein-1 (AP-1) by binding to its m1A subunit to release AP-1 from target membranes [93]. Since trafficking of the vesicular acetylcholine transporter between the synaptic vesicle membrane and the cytosol depends on AP-1, absence of PREPL could explain the reduced filling of synaptic vesicles with acetylcholine [93]. Mutations in the *PREPL* gene cause isolated PREPL deficiency [93]. So far, only a single patient with isolated PREPL-deficiency has been reported [93]. The female presented with congenital hypotonia, feeding difficulties, ptosis, and proximal muscle weakness. She later developed a waddling gait and used a walker [93]. LF-RNS did not evoke a decrement. The patient responded favorably to edrophonium and pyridostigmine.

##### SCN4A

*SCN4A* encodes for a post-synaptic sodium channel responsible for the generation of membrane action potentials. Phenotypically, mutations in this gene manifest in infancy with global hypotonia, impaired sucking, dysphagia, delayed postural and motor development and later in life with episodic, fluctuating muscle weakness like in periodic paralysis, bilateral facial palsy, ptosis, and ophthalmoparesis [94]. Episodes of periodic weakness could not be triggered by exercise, rest, potassium loading, or food, like in periodic paralysis [94]. In older patients, *SCN4A*-related CMS may manifest exclusively as easy fatigability [95]. In a 20yo normokalemic female, *SCN4A*-related CMS manifested as sudden attacks of respiratory and bulbar paralysis since birth, lasting 3–30 min and recurring one to three times per month, delayed motor development, easy fatigability, ptosis, ophthalmoparesis, and later as persisting facial, truncal, or limb weakness [96]. Some patients present with dysmorphism, such as high-arched palate, adduction deformity of the knees or ankles, and increased lumbar lordosis. Some patients are mentally retarded with cerebral atrophy on MRI [96]. RNS may be normal but higher stimulus frequency may trigger a decremental response [94]. AchEI are only marginally effective [94]. Acetazolamide together with potassium was ineffective [94].

##### RAPSN

*RAPSN* encodes for rapsyn, a post-synaptic membrane protein that anchors the nicotinic AchR to the motor endplate and also binds to β-dystroglycan [18]. Rapsyn is essential for clustering of the AchR at the post-synaptic membrane and required for the phosphorylation of CHRNB1 [18]. *RAPSN* mutations are a common cause of post-synaptic CMS [97]. The most common of the *RAPSN* mutation is N88G, but hetero-allelic mutations other than N88K can also occur [98, 99]. Occasionally, mutations in *RAPSN* become pathogenic only in case mutations in the *AK9* gene are simultaneously present [100]. Clinically, patients present with fluctuating ptosis, occasionally bulbar symptoms, neck muscle and mild proximal limb muscle weakness [97]. Infections can precipitate exacerbation of clinical manifestations [97]. In single patients prominent hyperlordosis can occur [101]. Usually, the response to AchEI is favourable but can be improved by adding 3,4 DAP [97]. Fluoxetine may worsen the decremental response in single patients [102]. In some patients general anesthesia may exacerbate muscle weakness [103]. The overall course is stable with intermittent worsenings [97].

##### PLEC1

*PLEC1* encodes for plectin, which crosslinks intermediate filaments to their targets in different tissues. The gene is ubiquitously expressed but manifests mainly in the skin, gastrointestinal tract, and the NMJ. The first patient with CMS due to a *PLEC1* mutation had early-onset muscular dystrophy and late-onset manifestations of a myasthenic syndrome (Table 1) [104]. RNS evoked a prominent decremental response [104]. AchEI (pyridostigmine) resulted in marked improvement of the muscular manifestations [104]. A second patient with epidermiolysis bullosa and CMS carried not only a *PLEC1* mutation but also a homozygous *CHRNE* mutation why it was difficult to decide to which degree the *PLEC1* mutation contributed to the CMS phenotype [105]. A third, Afro-American patient with epidermiolysis bullosa (EDB) developed myasthenic symptoms at age 39y [106]. RNS induced a decremental response already at age 15y. Histologically, NMJs showed destruction of the junctional folds and remodelling [106]. The patient died motionless at age 42y [106].

##### SLC25A1

*SLC25A1* encodes the mitochondrial citrate carrier across the inner mitochondrial membrane and is believed to be a key player in fatty acid and sterol biosynthesis, in chromosome integrity, and in autophagy regulation [107]. Missense mutations in the *SLC25A1* gene result in abnormal carrier function [107], hydroxyl-glutaric aciduria, and CMS. So far, CMS due to *SLC25A1* mutations has been reported in 3 English sibs. Two of them presented with easy fatigability and permanent weakness since early infancy [107]. One had had moderate intellectual disability [107]. Another developed obsessive convulsive tendencies and had pes cavus [107]. The third patient had a more severe phenotype with poor suck, hypotonia, apneas, optic atrophy, psychomotor delay, bulbar dysfunction, epilepsy, agenesis of the corpus callosum, hearing loss, and elevated urinary organic acids [107]. RNS was normal but SF-EMG revealed an increased jitter [107]. Only one of the three patients responded favourably to 3,4-DAP [107]. Pyridostigmine was ineffective in one case.

#### Glycosylation disorders

CMS may not only be due to mutations in genes involved in the structure and function of the motor endplate but also in genes involved in the glycosylation of proteins, lipids, or aglykones. Particularly glycosylation of AchR is impaired in CMS due to defective glycosylation. Glycosilation is essential for proper functioning of the NMJ and takes place in the endoplasmatic reticulum (ER) [108]. Currently, mutations in five genes are known that are involved in protein glycosylation and may be associated with CMS. These genes include *ALG2, ALG14, DPAGT1, GFPT1*, and *GMPPB* [109]. Though they are ubiquitously expressed, they predominantly manifest at the NMJ. Because of the clinical and histological findings the term “limb-girdle myasthenic syndrome with tubular aggregates” was coined.

##### GFPT1

*GFPT1* encodes for the glutamine-fructose-6-phosphate transaminase-1, which is a key rate-limiting enzyme that controls the flux of glucose into the hexosamine biosynthetic pathway, providing building blocks for the glycosylation of proteins and lipids [110]. *GFPT1* is ubiquitously expressed but it is not readily apparent why mutations in this gene cause symptoms restricted to the NMJ [110]. Mutations in *GFPT1* may lead to illegitimate binding of micro-RNAs resulting in reduced protein expression [111]. Patients manifest clinically with early-onset prominent LGMD-like weakness, easy fatigability and minimal cranio-bulbar symptoms [112, 113]. Muscle MRI may reveal T1-hyperintensities [26]. Maintenance of NMJs is dramatically impaired with loss of post-synaptic junctional folds and evidence of denervation-reinnervation processes affecting the three main NMJ components [112]. There may be mild reduction of the axon terminal size and post-synaptic fold simplification [114]. Most patients respond beneficially to AchEI [115]. In some patients the beneficial effect may be dramatic [116].

##### GMPPB

*GMPPB* encodes the catalytic enzyme GMPPB, which converts mannose-1-phosphate and GTP to GDP-mannose. GDP-mannose serves as a sugar donor [117]. The amount of protein may be hardly reduced [118]. *GMPPB* mutations manifest as mild, late-onset CMS. As with other glycosylation defects, ocular and facial muscles are largely spared and limb muscles are predominantly affected [109]. Muscle weakness may be fluctuating and associated with myalgias and calf hypertrophy [118]. Creatine-kinase (CK) is frequently elevated. RNS may be decremental, SF-EMG indicates a transmission defect, and EMG may be myogenic [118]. Muscle weakness in patients carrying *GMPPB* mutations is non-proportionally prominent compared to only mild abnormalities on EMG or muscle MRI [109]. On the contrary, muscle biopsy shows marked dystrophic features [119]. In a review of five patients carrying *GMPPB* mutations, four had dystrophic features with reduced labelling for alpha-dystroglycan [119]. Muscle MRI may show fatty degeneration of paraspinal, thigh adductor, and calf muscles or edema in the soleus muscle [120] or selective involvement of the calves in single cases [118]. Onset of clinical manifestations can be >70y of age [120]. Patients usually respond favourably to AchRI alone or in combination with 3,4-DAP and/or salbutamol [119].

##### ALG2

*ALG2* encodes the α-1,3-mannosyl-transferase that catalyses early steps in the asparagine-linked glycosylation pathway [108]. *ALG2* mutations result in severely reduced expression of *ALG2* in muscle [108]. Phenotypically, *ALG2* mutations manifest with infantile onset proximal muscle weakness, hypotonia, delayed motor milestones, and contractures [108]. Some patients may never achieve ambulation, some may develop bulbar symptoms. Severity and progression of the LGMD-like pattern muscle affection may be highly variable even within a single family [121]. RNS may be decremental. Muscle biopsy may reveal type-I-muscle fiber predominance or increased fibre-size variation [108]. Muscle biopsy may show myopathic features, ragged red fibers, and a sub-sarcolemmal accumulation of abnormally structured mitochondria [121].

##### ALG14

*ALG14* encodes a protein that is thought to form a multi-glycosyl-transferase complex with ALG13 and DPAGT1 to catalyse the first of two committed steps of asparagine-linked protein glycosylation [108]. Clinically, a rapidly progressive, early-onset and a benign late-onset form with variable clinical presentation can be delineated [108, 122]. The first two patients reported carrying *ALG14* mutations had adult-onset muscle weakness. Patients with early onset disease may present with a slightly different phenotype compared to patients with late-onset disease [122]. In a recent study of 5 early-onset patients, all presented with severe muscular hypotonia, progressive cerebral atrophy, and therapy-refractory epilepsy [122]. Three patients had congenital contractures [122]. In 2 patients, RNS was decremental. Treatment with AchEI led only to temporary improvement. All patients died during their first year of life [122].

##### DPAGT1

*DPAGT1* encodes for an essential ER-resident enzyme catalyzing the first committed step of N-linked protein glycosylation [123]. DPAGT1 is required for efficient glycosylation of AchR subunits and for efficient export of AchRs to the cell surface [123]. Accordingly, the number of AchRs is reduced [123]. Clinically, patients present with prominent LGMD-like weakness and minimal cranio-bulbar symptoms [67]. Isolated PREPL deficiency may go along with growth hormone deficiency and cystinurea [93]. Some patients present with intellectual disability and autistic features [124]. Single patients may exhibit restricted ocular abduction and long finger flexor contractions [125]. LF-RNS evokes a typical decrement [67]. Muscle MRI may reveal T1-hyperintensities [26]. Muscle biopsy in the advanced stage shows tubular aggregates [67], hypoplastic endplates, fiber-type dysproportion, and degeneration of muscle fiber organelles resulting in autophagocytosis [124]. Typically, AchEI and 3,4-DAP are effective [67]. Neostigmine reduced the decrement but pyridostigmine had no effect [124]. 3,4DAP improved the patient’s strength.

### Phenotypic heterogeneity and allelic variants

There are several proteins in which the same mutations may go along with phenotypic heterogeneity (allelic variants) [21, 120]. For example *GMPPB* mutations may mimic LGMD or congenital muscular dystrophy in cases in which dystrophic features are more prominent than CMS features [109]. In these patients the NMJ may be morphologically normal [109]. Mutations in *GMPPB* not only manifest as CMS but also as dystroglycanopathy [117]. *PLEC* mutations may not only cause CMS but also LGMD2Q, pyloric atresia, or epidermiolysis bullosa. Mutations in *SLC25A7* not only cause CMS but also AD forms of distal motor neuropathy [20]. Mutations in *DPAGT1* also cause congenital glycosylation-I defect and LGMD [18]. Additionally, there is intra- and inter-familial phenotypic heterogeneity despite the same genotype and a possible gender effect [14]. It is also important to mention that primary myopathies may go along with secondary transmission disease, which does not represent CMS, such as in patients with congenital myopathy due to *TPM2* mutations [126], or patients carrying mutations in *KLHL40, BIN1, DNM2, MTM1, TPM3*, or *RYR1*. Importantly, secondary transmission disease frequently responds beneficially to AchEI.

### Diagnosis

Diagnosing CMS relies on a thorough work-up by means of the history, clinical exam, blood tests, electrophysiological investigations, lung function tests, polysomnography, the tensilon test, eventually muscle biopsy, and the confirmation of a heterocygote or biallelic pathogenic variant in one of the 32 CMS genes. A CMS should be generally suspected if 1. there is easy fatigability or permanent weakness, most frequently in the ocular, facial, bulbar, axial, respiratory, or limb muscles with onset from birth to childhood; 2. the family history is positive for clinical manifestations of CMS; 3. history and clinical exam suggest myasthenia gravis but where AchR-, MUSK-, and LRP4-antibody tests are negative; 4. LF-RNS evokes a decrement of > 10% or if SF-EMG shows increased jitter or blockings; 5. clinical manifestations respond to AchEI; 6. there is a lack of improvement upon immunosuppressive therapy; 7. the family history suggests an AD/AR transmitted disease; 8. there is absence of a major pathology on muscle biopsy; and if 9. a specific syndrome (e.g. Escobar syndrome, Pierson syndrome (eye disease and nephropathy)) is present [24]. Mixing phenotype and age at onset, three phenotypes can be differentiated, which are the infantile-onset type, childhood-onset type, and the LGMD-type [6].

#### History

In case the history can be taken, patients may report easy fatigability, fluctuating or permanent weakness of ocular, bulbar, facial, axial, or limb muscles, double vision, ptosis, dysarthria, dysphagia, hypoacusis, head drop, or respiratory insufficiency. Patients may also recognise dysmorphism, they may report neuropathic pain, seizures, pterygia, contractures, hyperlaxity of joints, abnormal speech, cognitive impairment, respiratory insufficiency, or skeletal deformities.

#### Clinical exam

The neurological exam may be normal or abnormal.

##### Muscular features

Muscular abnormalities include ptosis, ophthalmoparesis, facial weakness, bulbar weakness (dysarthria, dysphagia), axial weakness (head drop, camptocormia), dyspnea, limb weakness, hypotonia, or reduced tendon reflexes. Rarely, patients may present with muscle wasting, particularly of limb muscles [81]. Atrophy of the skeletal muscles is particularly reported in *MUSK*-related CMS [81].

#### Non-muscle signs

##### Facial dysmorphism

There are a number of dysmorphic features that occur in specific CMS subtypes [54]. These include long face (*SYB1*) [7], hypertelorism (*SYB1*) [7], narrow jaw (*RAPSN*), saddle nose (*SYB1*) [7], and high-arched palate (*SCN4A*) [96]. In a Saudi female carrying a *COLQ* mutation, microcephaly was reported (Table 3) [41]. Microcephaly was also reported in *MUNC13–1*-related CMS.

**Table 3:** Typical clinical manifestations of CMS subtypes

Phenotypic feature	CMS subtypes
Myopathic	
LGMD-type	COLQ, DOK7, MUSK, GFPT1, ALG2, ALG14, DPAGT1
Respiratory insufficiency	SLC18A3, SYB1, COLQ, LAMB2, CHRNB1, CHRND, CHRNE, CHRNG, MUSK, NYO9A, LRP4, COL13A1, SCN4A, RAPS
Episodic apnea	CHAT, MUSK, SLC5A7, SLC25A1, RAPSN, COLQ
Head drop	AGRN
Myopathic EMG	CHRNB1, ALG2, PLEC1, GMPPB
Double response	CHRNE, COLQ, SCCMS, ACHE-deficiency, CHRNA1, CHRNB1, CHRND
Non-myopathic	
Cognitive dysfunction	SLC25A7, DPAGT1, SNAP25, COL13A1, MYO9A, CHRNB1, CHRND
Facial tics	LAMA5
Cerebral atrophy	SCN4A, ALG14
Agenesis of corpus callosu	SLC25A1
Epilepsy	ALG14, SLC25A1, MUNC13–1
Facial dysmorphism	SYB1, RAPSN, SCN4A, COLQ
Myopia	LAMA5
Hypoacusis	SLC25A1, SYT2
Vocal cord paralysis	COLQ, DOK7
Neuropathy	SYT2, SLC25A7
Arthrogryposis multiplex	SLC5A7, CHRNG
Contractures	SNAP25, VAMP1, CHRNA1, ALG2, ALG14, RAPSN, CHRND, CHRNG, CHAT
Scoliosis	COLQ, CHRNE, VAMP1
Hyperlordosis, hyperkyphosis	SCNA4, RAPSN, SYB1
Adduction deformity of knees	SCN4A
Cubitus valgus	PLEC1
Foot deformity	SYT2, RAPSN, CHRNG, SLC25A1, COLQ
Hyperlaxity of joints	SYT2, VAMP1, COL13A1
Cutaneous blisters	PLEC1
Pterygia	CHRNG
Systolic dysfunction	SLC18A3
Pierson syndrome	LAMB2
Cerebellar ataxia	SNAP25
Laryngospasm	SCN4A
Deterioration in cold water	SLC18A3
Hip dysplasia	SYT2
Cryptorchism	CHRNG
Arachnodactylia	CHRNG
Microcephaly	MUNC13–1

##### Skeletal abnormalities

Hyperlordosis or hyperkyphosis were reported in patients carrying *SCN4A* [97], *RAPSN* [101], or *SYB1* [7] mutations. Scoliosis may occur in *CHRNE*-related CMS [27] but also in *COLQ*-related CMS [43]. Foot deformities include pes cavus (hollow foot), pes planus, or hammertoes (*SYT2*-related CMS [127], *SLC25A1*). Club feet have been found in *RAPSN*-related CMS [54], and *COLQ*-related CMS [41]. In *SCN4A*-related CMS adduction deformity of the knees and ankles were reported [96]. Cubitus valgus was reported in *PLEC1*-associated CMS [106]. Hyperlaxity of joints and hip dysplasia may occur in *SYT2*-related CMS [32]. Joint laxity and kyphoscoliosis were reported in association with *VAMP1* [31] and *COL13A1* variants.

##### Cognitive impairment/neurodevelopmental delay

Cognitive dysfunction is only rarely a manifestation of a CMS phenotype. Mild to severe cognitive impairment has been reported in patients carrying mutations in the *SLC5A7* gene, *DPAGT1* gene [124], *SNAP25* gene [30], *COL13A1* gene [52], *MYO9A* gene [86], *MUNC13–1* gene, and in *SCN4A*-related CMS [20, 96]. In a study of 6 families, half of the probands carrying a *SLC25A7* mutation had mild cognitive impairment [20]. Recently, mutations in the *SLC18A3* gene have been shown to manifest as neurodevelopmental delay with cerebral atrophy [21]. Mutations in this gene may be also associated with infantile lethality [21]. Mild cerebral atrophy was reported in *SCN4A*-related CMS [96] and in *ALG14*-related CMS [123].

##### Neuropathy

Mutations in CMS genes, such as in *SYT2*, not only manifest in the skeletal muscle but also in the peripheral nerves as polyneuropathy [128]. Also *SLC5A7* mutations may manifest with distal neuropathy [21].

##### Epilepsy

There are a number of patients diagnosed with CMS who also had epilepsy. Epilepsy was reported in patients with CMS due to *SLC25A1* mutations [107], due to *MUNC13–1* mutations, or due to *ALG14* mutations [122].

##### Others

Cutaneous blisters of the dermis or the mucosa may be found in *PLEC1*-related CMS [106]. Agenesis of the corpus callosum and hearing loss have been reported in *MUNC13–1*-related [36] and *SLC25A1*-related CMS [107]. Two patients with *COLQ*-related CMS manifested with vocal cord paralysis [42]. Single patients with AchR-related CMS may develop pterygia. In *SLC18A3*-related CMS, systolic dysfunction was reported [29]. In a female with a *LAMA5*-variant, myopia and facial tics were described [51].

#### Blood tests

CK may be normal [81] or mildly elevated (maximally 10 times the upper limit) [1, 6], with the exception of *GMPPB*-related CMS. Antibodies against AchR, MUSK, or RLP4 are usually absent, being one of the diagnostic criteria for CMS [129].

#### Electrophysiologic evaluation

The most important electrophysiological investigation to support the CMS diagnosis are LF-RNS and HF-RNS. LF-RNS usually shows a decrement and only rarely an increment. If RNS is normal in two limb muscles, RNS of the facial muscles should be tried. HF-RNS usually shows an increment and only rarely a decrement [94, 130]. In patients carrying *SCN4A* variants, LF-RNS may be normal but may show a decremental response at higher stimulus rates [94]. Pre-synaptic CMS may not only be detected upon a profound decrement to LF-RNS but also by a prolonged period of post-activation exhaustion (decreased neuromuscular transmission on RNS after previous intense muscle contraction) [29]. In patients with *RAPSN*-related CMS, HF-RNS was followed by a decrement instead of the expected increment [130]. If RNS is normal, muscle contractions or exercise should be performed prior to the test. Instead of voluntary muscle contractions, 10 Hz stimulation during 5-10 min prior to LF-RNS may help to unmask an abnormal decrement or increment. In patients carrying *SYT1* mutations, CMAP amplitudes may be initially low but may markedly increase after forced exercise, like in Lambert-Eaton myasthenic syndrome [127]. If RNS is still normal, but still suspicion of a CMS, single fiber EMG is indicated, which may show increased jitter or increased number of blockings despite normal RNS [107]. Another test to unmask a NMJ defect is application of a single stimulus, which may be followed by a spontaneous second one (double response). The double response phenomenon can be typically observed in *COLQ*-related CMS and in SCCMS. In some patients needle-EMG may be myopathic [10]. Contrary to patients with periodic paralysis, myotonia may be absent on EMG in *SCN4A*-related CMS [94].

#### Lung function, polysomnography

Essential investigations to assess respiratory functions and to identify patients with nocturnal hypoventilation include lung function tests, arterial blood gas analysis, and polysomnography. Polysomnography is important to detect sleep disorders due to nocturnal apnoe/hypopnea recently reported in *COLQ*- and *RAPSN*-related CMS [131]. Symptoms indicative of nocturnal hypoventilation include daytime headache, restless sleep, impaired concentration, snoring, recurrent respiratory infections, or weight loss [6]. Applicability of lung function tests is restricted to cooperative patients. Non-cooperating patients may be investigated only by blood gas analyses and polysomnography.

#### Tensilon test

Though testing with edrophonium is frequently proposed, there are hardly reports about the details in patients with CMS. Generally, the test should be carried out only on an intermediate care unit (ICU) [6]. Initially, 2 mg should be applied, followed by another 2-5 mg in patients > 30 kg [6]. The dosage may be less in neonates and infants. The strongest effect will be achieved after 30s. Before the test, it is important to define an endpoint, such as ptosis, ophthalmoparesis, or weakness of limb muscles. Alternatively to edrophonium, pyridostigmine can be applied orally. Some patients with *CHRNE* mutations may show a striking response to the ice pack test [64].

#### Muscle biopsy

Muscle biopsy is normal in the majority of the cases. However, in glycosylation disorders due to mutations in the *GFPT1* gene tubular aggregates with synaptopathy and dramatic loss of post-synaptic functional folds and evidence of denervations/reinnervation processes affecting the three main NMJ components can be found [112]. In patients carrying *MUSK* mutations, increased fiber-size variability has been reported [81]. Patients with *COLQ*- or *GMPPB*-related CMS may show dystrophic features on muscle biopsy [45, 121]. Patients with *COLQ*- and *ALG2*-related CMS may show type-I-fiber predominance [45, 108].

#### Genetic testing

The most important investigations for diagnosing CMS are genetic tests. Different approaches for genetic testing can be applied, including single gene testing, multiple gene panel testing, or comprehensive genetic testing (WES, whole genome sequencing (WGS)) [6]. Single gene testing is indicated if a single gene accounts for a large proportion of the phenotype or if the phenotype and ancestry suggest a mutation in a particular gene as most likely. Sequencing of the gene of interest is carried out first, followed by gene-targeted deletion / duplication analysis [6]. Particular phenotypic features (apnea, non-response to AchEI, double response, increment on RNS, dysmorphism, foot deformities, neuropathy, epilepsy, contractures, AD/AR trait, or the ethnic origin (Maghreb, Roma, Spain/Portugal, Central/Western Europe) may guide the clinician to suspect a particular CMS subtype. For example, AD transmission is indicative of *SYT1-, SLC5A7-, SNAP25*-related, and SCCMS subtypes, usually present after adolescence with mild phenotypes. Only rare cases present in early life and become severely disabled in the first decade [18]. On the contrary, FCCMS usually present in early childhood with an infantile phenotype.

Due to the phenotypic heterogeneity, however, multi-gene panels are emerging as first-line diagnostic tool. If serial single gene testing or multi-gene panels fail to establish the diagnosis, WES should be considered [6].

#### Differential diagnoses

Differential diagnoses that have to be excluded before diagnosing CMS in adults include myasthenia gravis, motor neuron disease, including Kennedy disease, limb girdle muscular dystrophy, facio-scapulo-humeral muscular dystrophy, mitochondrial disorders, and hereditary neuropathies (Table 4). Myasthenia gravis usually has its onset in adulthood, however, when patients with myasthenia are young and sero-negative, differentiation from CMS can be challenging. Differential diagnoses that have to be excluded before diagnosing CMS in infants or children include transient neonatal myasthenia gravis, spinal muscular atrophy, congenital muscular dystrophy, congenital myotonic dystrophy-1, early-onset mitochondrial disorder, congenital myopathy, brainstem lesions, Moebius syndrome, and botulism (Table 4). Clinical phenotypes of CMS share significant overlap in their clinical presentations with mitochondrial disorders, leading to challenges in establishing the correct diagnosis [45].

**Table 4:** Differential diagnoses of CMS

Differential diagnosis	Early onset	Adult onset
FADS	yes	no
Periodic paralysis (SCNA4)	yes	yes
CDG	yes	no
Spinal muscular atrophy	yes	yes
Mitochondrial disorders	yes	yes
Myasthenia gravis	no	yes
Transient neonatal myasthenia	yes	no
Congenital muscular dystrophy	yes	no
Congenital myopathies	yes	no
Congenital myotonic dystrophy	yes	no
Brain stem abnormalities	yes	yes
Moebius syndrome	yes	no
(Infantile) botulism	yes	yes
Kennedy disease	no	yes
LGMD	yes	yes
FSH-MD	yes	yes
Hereditary neuropathies	yes	yes

*FADS* Fetal akinesia deformation sequence, *CDG* congenital disease of glycosylation, *LGMD* limb girdle muscular dystrophy, *FSH-MD* facio-scapulo-humeral muscular dystrophy

### Therapy

Therapy of CMS is not standardised due to the low number of patients and thus the lack of sufficiently powered treatment studies. Additionally, the genotypic and phenotypic heterogeneity makes it difficult to recruit homogenous groups required for treatment studies. Due to the rarity of CMS, therapy trials will meet the requirements for an appropriately designed treatment study only when applying an international, multicentre design. Generally, treatment may be classified as symptomatic or causal, invasive or non-invasive, or as established or experimental. Since no causal treatment for CMS is available, only symptomatic measures can be offered to these patients. Among the non-invasive symptomatic measures, drug treatment and non-drug treatment can be differentiated. Disadvantage of most reports is that dosages of agents applied, type of combinations, and duration of drug therapy are frequently not or insufficiently reported. There are also hardly reports available about side effects of the various agents applied.

#### Non-invasive symptomatic treatment

##### Drugs

There are several drugs available, which are applied to CMS patients but since some of them may exhibit severe side effects, these drugs need to be applied with caution until there is clear evidence that a particular patient profits from such compounds. Only in case of an emergency in a suspected CMS, drugs can be tried without previous genetic confirmation of the diagnosis.

##### AchE-inhibitors

AchEI are the drugs most frequently given to CMS patients (Table 5) but may not be effective in each of them (Table 5) [42]. AchEI may even deteriorate clinical manifestations in certain subtypes of CMS, such as in *COLQ-, LAMB2-, DOK7-, MUSK*-, or *LRP4*-related CMS. In case of an infection, prophylactic application of AchEI may be recommended. Prophylactic AchEI together with antibiotics may prevent the occurrence of episodic apnea and respiratory insufficiency.

**Table 5:** Effectiveness of drug treatment in the 32 CMS subtypes

CMS subtype	AchEI&	3,4-DAP	SALB	ALB	Ephed	Fluoxetine
Pre-synaptic						
SLC5A7	e	nr	e	nr	nr	nr
CHAT	pe	nr	nr	nr	nr	nr
SLC18A3	pe	nr	nr	nr	nr	nr
SNAP25	ie	e	nr	nr	nr	nr
VAMP1	e	nr	nr	nr	nr	nr
SYB1	pe	nr	nr	nr	nr	nr
SYT2	pe	e	nr	nr	nr	nr
MUNC13–1	pe	pe	nr	nr	nr	nr
Synaptic						
COLQ	ie	pe	e	nr	e	nr
LAMB2	ie	pe	e	nr	e	nr
LAMA5	e	e	nr	nr	nr	nr
COL13A1	ie	e	e	nr	nr	nr
Post-synaptic						
CHRNA1	e/pe	nr	nr	nr	nr	nr
CHRNB1	ie	nr	nr	nr	pe	e
CHRND	pe	e	nr	nr	nr	nr
CHRNE	e/ie	ie	e	e	nr	e
CHRNG	nr	nr	nr	nr	nr	nr
FCCMS	e	e^a^	nr	nr	nr	nr
SCCMS	nr	nr	nr	nr	nr	e
DOK7	ie	nr	e	e	e	nr
MUSK	ie	ie/e	e	pe	ie	nr
MYO9A	e	e^a^	nr	nr	nr	nr
AGRN	ie	nr	nr	nr	e	nr
LRP4	ie	nr	nr	e	nr	nr
PREPL	e	nr	nr	nr	nr	nr
SCN4A	pe	nr	nr	nr	nr	nr
RAPSN	e^b^	e	nr	nr	e	nr
PLEC1	e	nr	nr	nr	nr	nr
SLC25A1	ie	e	nr	nr	nr	nr
Glycosilation defect						
GFPT1	e	nr	nr	nr	nr	nr
GMPPB	e	e^a^	e	nr	nr	nr
ALG2	e	nr	nr	nr	nr	nr
ALG14	pe	nr	nr	nr	nr	nr
DPAGT1	e	e	nr	nr	nr	nr

SALB: salbutamol, ALB: albuterole, Ephed: ephedrine, pe: partially effective, nr: not reported, e: effective, ie: ineffective, ^a^: in combination with AchEI, ^b^: in combination with 3,4-DAP, &: in *COLQ-, LAMB2-, DOK7-, MUSK*-, and *LRP4*-related CMS AchEI may deteriorate the clinical manifestations

##### 4-Diaminopyridine

The most frequently applied alternative drug to AchEI or the one most frequently given in combination with AchEI is 3,4-DAP. 3,4-DAP increases the amount of acetylcholine released to the synaptic cleft. Additionally, it prolongs the presynaptic action potential. 3,4-DAP is not only effective in pre-synaptic but also in post-synaptic CMS [129]. 3,4-DAP may have only a mild beneficial effect in *COLQ*-related or *LAMB2*-related CMS (Table 5) [42]. 3,4-DAP may be ineffective in *CHRNE*- or *MUSK*-related CMS (Table 5). 3,4-DAP may be detrimental in FCCMS due to AR loss-of-function mutations and should be avoided in these conditions [12].

##### Salbutamol

Salbutamol is a β2-mimetic, which has been reported beneficial in *SLC5A7-, COLQ*-, *CHRNE-, DOK7-, MUSK-, COL13A1-,* and *GMPPB-*related CMS (Table 5) [14, 48].

##### Albuterol

Albuterol is a bronchodilator and an alternative to ephedrine and has a beneficial role in *CHRNE*- and *MUSK*-related CMS (Table 5).

##### Ephedrine

Ephedrine is an alkaloid from the group of phenyl-ethyl-amines, originating from the plant ephedra. It is used in medicine as a sympathomimetic agent, for asthma, as a decongestant, and in ophthalmology as a supplement of atropine. Ephedrine is usually well tolerated. It has been reported effective in *COLQ-, LAMB2-, DOK7*-, and *AGRN*-related CMS (Table 5). In a patient with *COLQ*-related CMS manifesting as vocal cord paralysis, hypotonia, ptosis, ophthalmoparesis, and facial diplegia, ephedrine was highly effective [42]. Ephedrine was ineffective in *MUSK*-related CMS [85].

##### Fluoxetine

Reports about the effect of fluoxetine in CMS are conflicting. While a beneficial effect has been reported in *CHRNB1* and *CHRNE*-related CMS (Table 5), fluoxetine worsened the clinical manifestations in *MYO9A*- and *RAPSN*-related CMS. Fluoxetine has been reported beneficial for muscle weakness in patients with SCCMS [132].

##### Others/experimental

Only single reports are available about the effect of acetazolamide, quinidine, and atracurium. Recently, zonisamide has been shown beneficial in experimental CMS, being attributed to its nerve-sprouting activity [133].

##### Non-drug treatment

Non-invasive, non-drug treatment relies on physiotherapy, speech therapy, and occupational therapy. To warrant mobility, orthoses, walkers, or wheel chairs may be used. Generally, patients with CMS should avoid strenuous exercise or infections, which may exacerbate symptoms of a transmission disease. NIPPV either during the night or the entire day may support insufficient self-breathing. In animals, antisense oligonucleotides (AONs) have been shown to be beneficial in *CHRNA1*-related CMS [53].

#### Invasive measures

In case of dysphagia, failure-to-thrive, or a nutritional disturbance, implantation of a PEG may be necessary. In case of respiratory insufficiency without the possibility of a NIPPV, intubation and mechanical ventilation may be indicated. Severe, symptomatic scoliosis may require surgical spinal correction [49]. Foot deformities may require surgical corrections.

#### Pregnancy and CMS

Pregnancy has been reported to exacerbate clinical manifestations of CMS [134]. In a study of 17 pregnancies of females from 8 families with a CMS it turned out that pregnancy deteriorated clinical manifestations of CMS [135]. In most cases, affected females recovered to the status quo ante within six months postnatally [135]. The outcome of neonates born to females with CMS is fair except in females with SCCMS [135]. To warrant a good outcome from pregnancy, close neurological surveillance is necessary.

### Prognosis and outcome

Prospective outcome studies are not available but in several observational studies, case studies, and case reports, the outcome has been mentioned. In a study of 79 CMS patients, those carrying a *DOK7* mutation had the worst outcome [134]. Among the 8 patients being wheel-chair-bound and ventilated, 6 carried a *DOK7* variant [134]. Since the clinical presentation is highly variable, also the outcome and prognosis may vary considerably between the various CMS types. The outcome is further influenced by acute deteriorations due to infections, fever, or psychosocial stress.

## Conclusions

Currently, 8 pre-synaptic, 4 synaptic, 15 post-synaptic, and 5 glycosilation defects are known to cause CMS. The most frequently reported CMS subtypes are *COLQ-, CHRNE-, RAPSN-, DOK7*-, and *CHAT*-related CMS (Table 1). Though CMS are congenital in the majority of the cases, it becomes increasingly evident that in some subtypes the onset may be in early or even late adulthood. However, severity of the disease is usually more pronounced in early-onset subtypes. Due to their intra- and inter-familial phenotypic heterogeneity, CMSs can be easily mixed up with other neuromuscular disorders, particularly LGMDs and mitochondrial disorders. A number of promising proposals have been launched for the treatment of certain CMS subtypes during recent years. They should be further evaluated to find out if they are also effective in other CMS subtypes. These measures include the application of a gene therapy with AONs and the application of zonisamide, which may trigger axonal sprouting. Whenever patients with myasthenic symptoms do not present with AchR- or MUSK-antibodies, do not respond to immunosuppressive treatment, have a positive family history for their phenotype, and show impaired neuromuscular transmission upon RNS or SF-EMG, a CMS should be considered.

## Abbreviations

AchEI: Acetylcholine-esterase inhibitors
AchR: Acetylcholine-receptor
AD: Autosomal dominant
AON: Antisense-oligonucleotide
AR: Autosomal recessive
CMAP: Compound muscle action potential
CMD: Congenital muscular dystrophy
CMS: Congenital myasthenic syndrome
DAP: Diaminopyridine
DNA: Desoxiribonucleic acid
EDB: Epidermiolysis bullosa
EMG: Electromyography
ER: Endoplasmatic reticulum
FADS: Fetal akinesia deformation sequence
ICU: Intermediate care unit
LF-RNS: Low-frequency RNS
LGMD: Limb girdle muscular dystrophy
MRI: Magnetic resonance imaging
NIPPV: Non-invasive positive pressure ventilation
NMJ: Neuromuscular junction
PEG: Percutaneous entero-gastrostomy
RNS: Repetitive nerve stimulation
SF-EMG: Single-fiber electromyography
VAchT: Vesicular acetylcholine transporter
WES: Whole exome sequencing
WGS: Whole genome sequencing
